# Intuitive stent simulation with internal cavity airway models created by a consumer-grade three-dimensional printer: A case series

**DOI:** 10.1016/j.rmcr.2025.102167

**Published:** 2025-01-17

**Authors:** Naoya Kitamura, Toshihiro Ojima, Yoshifumi Shimada, Keitaro Tanabe, Ryo Yokoyama, Naru Kitade, Koichiro Shimoyama, Tomoshi Tsuchiya

**Affiliations:** a Department of Thoracic Surgery, Toyama University Hospital, 2630, Sugitani, Toyama City, Toyama 930-0194, Japan; b Department of Thoracic Surgery, Kurobe City Hospital, 1108-1, Mikkaichi, Kurobe City, Toyama 938-8502, Japan

**Keywords:** 3D printing, Airway stenting, Airway model, Simulation, Silicon stent

## Abstract

Airway stenting, which can cause life-threatening complications, requires advanced techniques as well as safe and reliable procedures. Learning effective techniques and accurately identifying and simulating malignant airway stenosis before the procedure are crucial. However, traditional methods that use three-dimensional (3D) computed tomography images displayed on two-dimensional screens have limitations in accurately visualizing and simulating airway conditions. To address these issues, this study presents the creation of 3D airway models using a consumer-grade 3D printer. These cost-effective and time-efficient models replicate the airway lumen, allowing precise stent placement simulations and enhancing the learning process of airway stenting techniques. In a case series of five patients with malignant airway stenosis, these models facilitated effective stent placement, enhanced the understanding of airway anatomy, and contributed to procedural success. This study's findings suggest that the use of 3D airway models has substantial potential for broader clinical applications in airway stenting. However, evidence for the widespread adoption of this technology is insufficient, and further research is needed.

## Highlights

• Developed an economical, hollow airway model using a consumer-grade 3D printer.
• The model provided a realistic simulation of airway stenting for better skill gain.
• Internal cavity design allows intuitive understanding and precise stent placement.
• Enables preoperative simulation and education on malignant airway stenosis management.
• The method is quick, reproducible, and reduces the cost of medical modeling.

## 1 Introduction

Airway stenting requires a high level of skill by the respiratory physician because a momentary error can lead to patient death due to asphyxia. According to the past European and American guidelines, at least 10–20 procedures should be performed under the supervision of an experienced specialist, followed by 5–10 cases per year to maintain skill proficiency [1,2]. However, recent guidelines emphasize that airway stenting should be performed by experienced respiratory specialists but do not specify the number of cases or level of experience required to maintain skill proficiency [3]. This may be because airway stenting is becoming a less frequent procedure; hence, it has become difficult to obtain the same level of case experience as was once recommended. Therefore, it is considered an important issue to acquire and pass on skills efficiently even in a small number of cases.

Another important factor is the preoperative understanding and precise simulation of stenosis, particularly malignant airway stenosis. Three-dimensional (3D)-computed tomography (CT) technology has made it easier to identify airway stenosis than conventional methods of confirming airway stenosis using CT images alone [4]. However, it is sometimes difficult to accurately determine the extent of airway stenosis and the branching angle of the bronchus using only 3D images displayed on a two-dimensional screen or to simulate a complete view when a trimmed stent is applied.

To overcome these problems, a 3D printer has been used to create an airway model, and its usefulness in airway stenting has been reported. This technique facilitates the understanding of airway stenosis [5,6] and is effective in education [7]. In certain cases, 3D-printed T-tubes or silicone stents are directly implanted in patients with consent [8,9]. However, this technology remains in its developmental stage and is not widely used because of the high cost of creation, difficulty in creating internal cavity models, time required for creation, and other issues [10].

We have been developing a 3D airway model using a consumer-grade 3D printer to acquire and disseminate effective techniques for airway stenting and to facilitate highly accurate simulations. Our 3D airway model can be easily created using a consumer-grade 3D printer, and a hollow model can be created swiftly at a low cost [11]. Here, we present a method for creating airway models using this method, describe a case series of airway stent placement, and report the usefulness of airway models using a consumer-grade 3D printer.

## 2 Material and methods

In all five cases presented here, airway models were created and simulated using the following methods, after which stents were implanted.

### 2.1 3D airway modeling

**Fig. 1:**
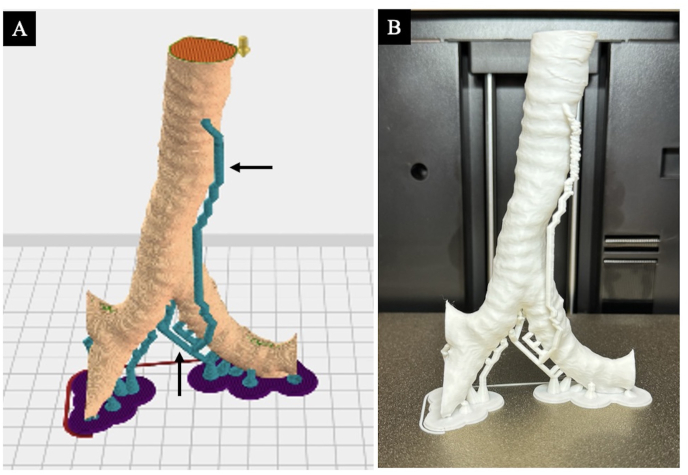
Process of creating the three-dimensional (3D) airway models. (A) Image of a 3D airway model created using FlashPrint^R^ (Flashforge, Zhejiang Province, China). The anchors are automatically designed to prevent the model from collapsing during the layering process (black arrows). (B) The 3D airway model was created using a consumer-grade 3D printer, the Flashforge Dreamer^R^ (Flashforge, Zhejiang Province, China).

CT images of the patients were imported into OsiliX MD(Pixmeo SARL, Geneva, Switzerland)—a Digital Imaging and Communications in Medicine viewer—for data conversion, and the lumen from the trachea to the bronchus was extracted as a region of interest (ROI). The images processed using OsiliX MD were imported into FlashPrint (Flashforge, Zhejiang Province, China)—which is a slicing software for 3D printers—to design the airway model for actual creation (Fig. 1A). These data were then imported into a 3D printer, the Flashforge Dreamer (Flashforge), and the flexible filament FilaFlex (FilaPrint, Florence, Italy) or TPU 3D Printer Filament (Overture, Wilmington, Delaware, United States) was used as a material to create a 3D airway model (Fig. 1B).

### 2.2 Simulation and trimming of the stent

**Fig. 2:**
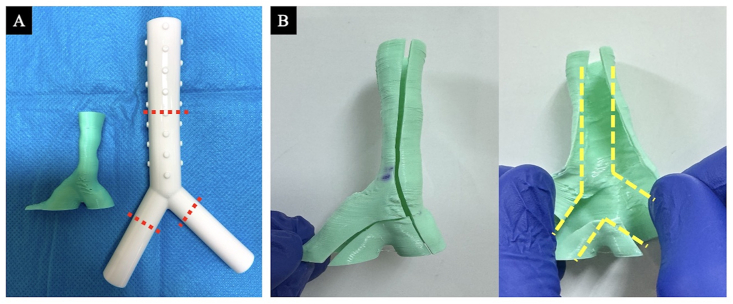
Stent simulation and trimming method. (A) The silicone stent is lined up next to the airway model and cut to a size that minimally covers the malignant airway stenosis site (red dotted line). (B) The wall of the airway model is cut open, and the trimmed silicone stent is loaded into the model if necessary (yellow dotted line). Then, the extent of stenosis coverage and its relationship to the branches (especially the right upper lobe) are verified for adequacy.

The 3D airway model created using the method described in 2-1 was brought to the operating room. The airway model and silicone stent were placed side by side or stacked, and the silicone stent was cut (trimmed) to a size that adequately covered the malignant airway stenosis site (Fig. 2A). If necessary, the airway model was cut open, and the trimmed silicone stent was loaded into the model to verify adequate coverage of the malignant airway stenosis site and its relationship with the branches (right upper lobe branch, especially for Y-shaped stents) (Fig. 2B). This information was shared with the thoracic surgeons, anesthesiologists, nurses, and radiologists in the operating room.

### 2.3 Placement of the airway stent

The anesthesiologist administered sedation without administering muscle relaxants, leaving the patient to breathe spontaneously. A rigid bronchoscope was placed in the trachea to observe the airway. If necessary, tumor cauterization with argon plasma coagulation, debulking, or airway dilatation with a balloon was performed, followed by the placement of a trimmed silicone stent under fluoroscopic guidance.

### 2.4 Ethics

The study used only existing data, and the participants’ personal information was completely anonymized; therefore, informed consent was deemed unnecessary.

## 3 Case Presentations

Patient clinical characteristics of cases 1–5 are presented in Table 1.

**Table 1:** Patient clinical characteristics.

Cases	Age	Sex	PS	Chief complaint	Diagnosis	Main airway stenosis sites	Type of silicone stent	Operating time (min)	ECMO	Stent migration	Complications	Survival time (months)
1	82	M	1	Asymptomatic	Esophageal cancer	Trachea	Y-shaped	63	–	–	–	8
2	70	M	3	Anorexia	Esophageal cancer	Trachea	Y-shaped	115	+	–	–	6
3	73	M	1	Dysphagia and dyspnea	Esophageal cancer	Trachea	Straight	53	–	–	–	5
4	67	M	1	Asymptomatic	Lung cancer	Rt. main bronchus	Y-shaped	81	–	–	–	2
5	75	F	1	Dyspnea	Lung cancer	Rt. main bronchus	Y-shaped	165	–	–	–	2.5

PS, Performance status; ECMO, Extracorporeal membrane oxygenation; F, Female; M, Male; Rt., Right.

### 3.1 Case 1

**Fig. 3:**
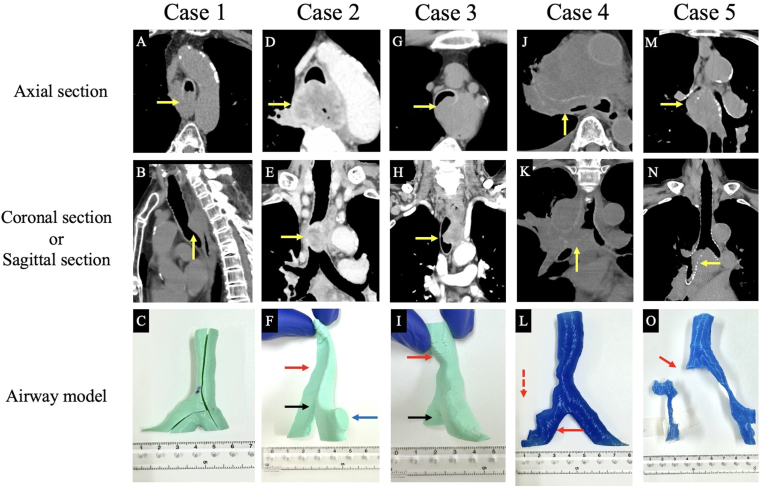
Chest computed tomography (CT) images of all cases and the airway models created. (A and B) Chest CT images of Case 1, showing malignant airway stenosis of the trachea due to metastatic lymph node infiltration from the membranous side of the trachea. (C) A slit was made in the airway model of Case 1, the trimmed stent was inserted, and a simulation was performed. (D and E) CT images of Case 2, showing esophageal cancer invasion from the membranous side of the trachea and malignant airway stenosis of the trachea. (F) An image of the airway model of Case 2 observed from the right dorsal side. The relationship between the tracheal bifurcation (black arrow), the stenosis caused by the tumor (red arrow), and the right upper lobe branch (blue arrow) can be understood. (G and H) CT images of Case 3, showing esophageal cancer invasion from the left side of the membranous part of the trachea and severe malignant airway stenosis of the trachea. (I) An image of the airway model of Case 3 observed from the right dorsal side. The distance between the tracheal bifurcation (black arrow) and the stenosis caused by the tumor (red arrow) is easy to understand. (J and K) CT images of Case 4, showing extensive malignant airway stenosis from the right main bronchus to the right upper lobe. (L) The airway model of Case 4 is depicted with a slight opening in the right main bronchus (red arrow); however, the right upper lobe branch is not depicted owing to occlusion (red dashed arrow). (M and N) Chest CT images of Case 5, showing malignant airway stenosis in the right main bronchus. (O) The actual airway model of Case 5 has a discontinuity (red arrow) because the right main bronchus cannot be depicted owing to the malignant airway stenosis. (All yellow arrows indicate tumors that could potentially cause malignant airway stenosis.)

An 82-year-old male was treated with nivolumab for esophageal cancer. However, metastatic recurrence in the mediastinal lymph node and tracheal malignant airway stenosis were observed (Fig. 3A and B). The best supportive care (BSC) was decided because no treatment options were available. Although he had no subjective symptoms, a Y-shaped silicone stent was implanted to prevent airway malignant airway stenosis, with reference to the airway model (Fig. 3C). The stent was implanted after dilatation with a balloon owing to the risk of damage to the membranous part of the trachea. The patient was discharged on postoperative day 5 but died 8 months postoperatively due to worsening disease.

### 3.2 Case 2

A 70-year-old male presented with a chief complaint of anorexia. He had advanced esophageal cancer (cT4bN1M0, stage IVA) and tracheal malignant airway stenosis due to the presence of a tumor (Fig. 3D and E). Because the malignant airway stenosis was extremely severe and there was concern about asphyxia during the procedure, a Y-shaped silicone stent was placed in combination with venovenous extracorporeal membrane oxygenation. This stent was trimmed to the optimal shape based on the airway model (Fig. 3F). Radiochemotherapy was administered to shrink the tumor; however, the patient died 6 months postoperatively.

### 3.3 Case 3

A 73-year-old male presented at our hospital with dysphagia and dyspnea. The patient had advanced esophageal cancer (cT4bN2M0, stage IVA) and tracheal malignant airway stenosis caused by the presence of a tumor (Fig. 3G and H). We decided to place a stent because the patient had marked respiratory distress, to the point where he could not lie in the supine position. After creating and evaluating the airway model (Fig. 3I), it was determined that the distance from the tracheal bifurcation to the tumor was long, and a Y-shaped silicone stent would have provided an overly large coverage area. Therefore, a straight silicone stent was placed. The patient's symptoms improved, and he was treated with radiochemotherapy; however, he died 5 months postoperatively.

### 3.4 Case 4

A 67-year-old male was diagnosed with a right upper lobe tumor upon close examination of an abdominal mass. He was diagnosed with right upper lobe lung cancer (cT4N2M1, stage IVB) and was initiated on radiotherapy. However, the tumor had invaded the right main bronchus and right upper lobe branch, resulting in progressive malignant airway stenosis (Fig. 3J and K). Respiratory distress also worsened; therefore, the tumor was resected as much as possible under rigid bronchoscope, and a Y-shaped silicone stent was implanted, with reference to the airway model (Fig. 3L). The right upper lobe branch was sacrificed owing to obstruction caused by tumor invasion, and the right leg of the stent was opened to the middle trunk. Three courses of carboplatin, pemetrexed, and pembrolizumab were subsequently administered, but the disease control was difficult; hence, BSC was adopted. The patient died 4 months postoperatively.

### 3.5 Case 5

A 75-year-old female presented with a chief complaint of dyspnea. Chest CT revealed mediastinal lymph node enlargement at the tracheal bifurcation and airway narrowing due to invasion of the right bronchus (Fig. 3M and N).

The tumor was resected as much as possible, the right main bronchus was fully opened, and the right leg of the stent was placed to open toward the right upper lobe branch, with reference to the airway model (Fig. 3O). Dyspnea improved with stent placement, and the patient was diagnosed with non-small cell lung cancer (programmed cell death ligand 1, 90–100 %). Although she was treated with radiation therapy (36 Gy) and pembrolizumab, she died 2.5 months postoperatively owing to the deterioration of her general condition caused by complications such as pneumonia and pleural effusion.

## 4 Discussion

The 3D models of the airway created using a consumer-grade 3D printer presented in this study may play an important role in airway stenting from various perspectives. A realistic model created by a 3D printer facilitates the learning of the technique before treating a patient in the clinical setting. Techniques that are difficult to explain using language alone, such as the appropriate distance to advance the applicator and the method for extruding and implanting the silicone stent, can be learned directly using the model.

**Fig. 4:**
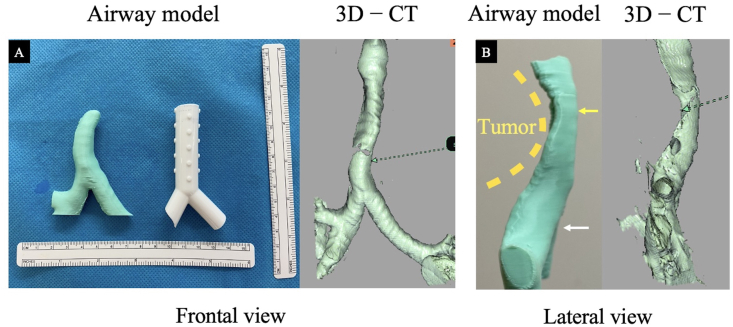
Comparison of the airway model and 3-dimensional (3D)-computed tomography (CT) images. (A) Comparison of the airway model, trimmed stent, and 3D-CT image in the frontal view showing that the airway model has almost the same shape as the 3D-CT image and that the silicone stent can be cut precisely to allow proper opening of the silicone stent into the right upper lobe. (B) The airway model in the lateral view is comparable to the 3D-CT image, and the distance between the tracheal bifurcation (white arrow) and the malignant airway stenosis site (yellow arrow) is easy to understand.

The model is also an excellent tool in terms of understanding the airway stenosis site and simulation. The model created by the consumer-grade 3D printer reproduces the airway condition almost identical to the 3D-CT image while allowing the user to hold it in their hands and intuitively understand it (Fig. 4A and B), thus enabling a deeper understanding of airway stenosis and the lesion location [7]. In particular, silicone stents can rarely be implanted straight off the shelf, and trimming is essential to ensure a good fit in patients of different body sizes and conditions. Gildea et al. reported two cases of implantation of 3D-printed tailor-made stents with a special United States Food and Drug Administration approval [9]; however, the long-term risks of keeping a medical device that has not been formally approved in a body are uncertain. The widespread adoption of this approach for patients with urgent needs, such as those with airway stenosis due to malignancy is likely to require more time. Therefore, making good use of existing products remains crucial. Important points for Y-shaped silicone stents include maintaining proper opening to the right upper lobe branch—which is a short distance from the tracheal bifurcation—and making the stent long enough to prevent migration but not to interfere with expectoration [12]. In this context, the ability to operate with the same sensation as that of the actual trachea to determine the appropriate angle and length may help shorten the operative time, increase the success rate of the procedure, and reduce complications.

**Fig. 5:**
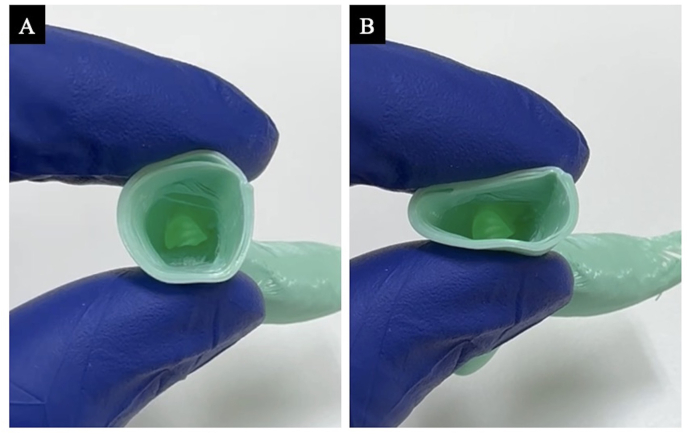
Flexibility of the airway model. A comparison of the airway model in its natural state (A) and under compression (B) shows the flexibility and strength of the airway model.

**Fig. 6:**
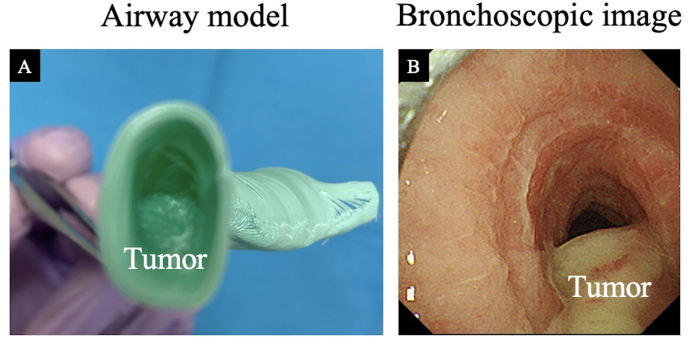
Comparison of the airway model and bronchoscopic findings. Airway model (A) and actual bronchoscopic findings (B) in Case 1. The airway model accurately depicts the actual airway.

As previously reported [11], the 3D airway model using our method is inexpensive and quick to create, and it is characterized by a soft structure with an internal cavity (Fig. 5A and B). The airway model can be produced with reproducibility equivalent to bronchoscopic findings of the airway (Fig. 6A and B). Previous studies have reported problems such as high cost, the fact that only internally enriched models can be made in which the state of the airway lumen is not visible, and the fact that it takes several days to create a model [10]. However, our technique using a consumer-grade 3D printer (the model used in this study cost less than $695) can create a model within 1–2 hours, the filament material used is inexpensive ($0.14–$0.21 per model), and the airway lumen constriction can be dependably reproduced. This enables the creation of high-quality models while maintaining low costs, thus facilitating their dissemination and promoting their use in clinical practice.

This study has one limitation. If the bronchus is completely stenosed, as in Cases 4 and 5, the model may be interrupted, raising concerns about the simulation of stent length. However, if a past CT scan without occlusion is available, a mold can be created based on that image. In cases where no past CT scan is available, a mold can be created by setting an ROI on the bronchial wall, allowing for the creation of a model without interruption, even in complete stenosis. However, in this case, a possibility that the extent of the stenosis may not be accurately captured exists; hence, the method for creating the model will need to be determined on a case-by-case basis.

The applicability of 3D printers prompted by recent technological innovations has been suggested; however, owing to the scarcity of cases, its use in clinical practice remains insufficient in terms of evidence building. The usefulness of the airway model and its development will likely require further research.

## 5 Conclusions

The 3D airway model using a consumer-grade 3D printer is useful for acquiring and simulating airway stenting techniques, and it has excellent potential for clinical application because it can be created in a short time and at a low cost. However, further evidence is needed and additional research is required before it can be routinely used in the clinical setting.

## CRediT authorship contribution statement

**Naoya Kitamura:** Writing – review & editing, Writing – original draft, Investigation, Data curation. **Toshihiro Ojima:** Writing – review & editing, Validation, Investigation, Conceptualization. **Yoshifumi Shimada:** Writing – review & editing, Investigation. **Keitaro Tanabe:** Writing – review & editing, Investigation. **Ryo Yokoyama:** Writing – review & editing, Investigation. **Naru Kitade:** Writing – review & editing, Investigation. **Koichiro Shimoyama:** Writing – review & editing, Investigation. **Tomoshi Tsuchiya:** Writing – review & editing, Validation, Supervision.

## Data statement

Not applicable.

## Funding

This research did not receive any specific grants from funding agencies in the public, commercial, or not-for-profit sectors.

## Declaration of competing interest

The authors declare that they have no known competing financial interests or personal relationships that could have appeared to influence the work reported in this paper.
